# *Ruminococcus gnavus* and Biofilm Markers in Feces From Primary Bile Acid Diarrhea Patients Indicate New Disease Mechanisms and Potential for Diagnostic Testing

**DOI:** 10.1016/j.gastha.2025.100712

**Published:** 2025-05-27

**Authors:** Evette B.M. Hillman, Danielle Carson, Julian R.F. Walters, Martin Fritzsche, Ryan Mate, Katie E. Chappell, Elena Chekmeneva, Maria Gomez Romero, Stephen J. Lewis, Sjoerd Rijpkema, Elizabeth M.H. Wellington, Ramesh Arasaradnam, Gregory C.A. Amos

**Affiliations:** 1 Diagnostics, Medicines and Healthcare products Regulatory Agency, London, UK; 2 School of Life Sciences, The University of Warwick, Coventry, UK; 3 Division of Digestive Diseases, Imperial College London, London, UK; 4 Imperial College Healthcare Trust, London, UK; 5 The National Phenome Centre, Imperial College London, London, UK; 6 Department of Gastroenterology, University Hospital Plymouth, Plymouth, UK; 7 Peninsula Medical School, University of Plymouth, Plymouth, UK; 8 Department of Gastroenterology, University Hospitals Coventry & Warwickshire, Coventry, UK; 9 Warwick Medical School, The University of Warwick, Coventry, UK

**Keywords:** Chronic Diarrhea, Mucosal Biofilms, Iso-Bile Acids, Microbiome, Bile Acids

## Abstract

**Background and Aims:**

Bile acid diarrhea (BAD) is a common cause of frequent loose stools, urgency, and incontinence, which is under-recognized due to limited diagnostic test availability and unclear pathogenesis. This study aimed to investigate fecal changes in well-defined subjects.

**Methods:**

Fecal samples were compared in BAD patients (n = 26), diagnosed by SeHCAT testing, and healthy controls (n = 21). Shotgun metagenomic sequencing was used to identify microbiome species and functional genes. An extended set of 38 bile acids was quantified by liquid chromatography mass spectrometry, including various epimers and intermediates, such as iso- (3-beta-OH), oxo (keto), allo (5-alpha), and 3-sulfated forms.

**Results:**

Alpha diversity, reflecting microbial richness, was reduced in BAD patients with severe forms of the disease, while beta diversity demonstrated distinct microbial profiles between groups. *Ruminococcus gnavus* (*R. gnavus*) was prevalent in BAD patients but rare in controls (odds ratio = 73), while *Firmicutes bacterium* CAG110, *Eubacterium siraeum* and 2 *Oscillibacter* species were less common in BAD (odds ratios = 25–30). Overall, 99 taxa differed significantly between groups. Bile acidtransforming genes (*baiA*, *baiB*, *hdhA*) were more abundant in BAD samples (*P* ≤ .0012). Most fecal bile acids, including iso-bile acids and intermediates, were higher in BAD. Elevated ursodeoxycholic acid-3-sulfate and relatively lower lithocholic acid and allo-bile acids, including isoallolithocholic acid, reflect changes in bacterial metabolism. Biofilm-associated genes (*bssS*, *pgaA*, *pgaB*) were markedly elevated in BAD patients (*P* ≤ .00008). SeHCAT values negatively correlated with *R. gnavus* (rho −0.53, *P* = .008) and positively with *Eubacterium siraeum* (rho 0.41, *P* = .041).

**Conclusion:**

BAD may result from an overgrowth of *R. gnavus*, associated with intestinal biofilms and an altered bile acid metabolism.

## Graphical abstract

**Figure:**
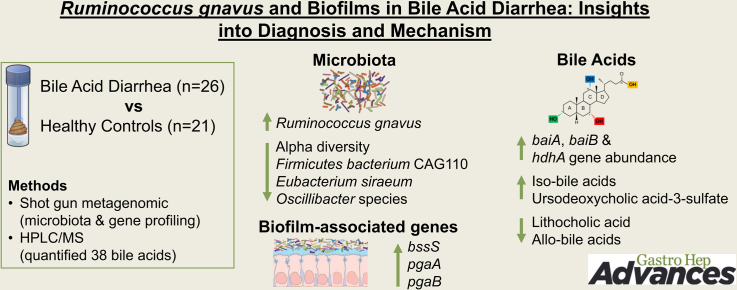

## Introduction

Bile acid diarrhea (BAD) is a common gastrointestinal disease, estimated to have a population prevalence of over 1%.^1^ Primary BAD (PBAD) is often misdiagnosed as diarrhea-predominant irritable bowel syndrome (IBS-D), and 25%–33% of people with IBS-D actually have BAD.^1^^,^^2^ Although this condition is not life threatening, it impairs the quality of life, leaving individuals housebound as a result of frequent watery diarrhea, urgency, incontinence, as well as bloating and abdominal pain.^3^

High levels of bile acids in the colon cause excess water secretion and increased motility, leading to symptoms. The excess bile acids may result from bile acid malabsorption from ileal disease, surgery, or other gastrointestinal disorders, causing secondary BAD. More commonly, it is idiopathic, PBAD, where no impairment of absorption has been found.^4^ The causes of PBAD remain unclear but include impaired feedback regulation by ileal fibroblast growth factor (FGF19), leading to excessive bile acid synthesis,^5^ and associations with certain genetic polymorphisms.^6^

The gold standard for diagnosing BAD is the selenium-75-homocholic acid taurine (SeHCAT) nuclear medicine test measuring 7-day bile acid retention and loss.^7^ However, SeHCAT is not widely used or licensed in many countries (including the United States), and even where it is available, recognition of BAD as a possible diagnosis of unexplained chronic diarrhea is often delayed for many years.^3^ Other diagnostic tests include measurements of total or primary bile acids in feces and plasma 7α-OH-4-cholesten-3-one (C4) as a marker of new bile acid synthesis.^8^ SeHCAT testing was shown in a recent trial to be a better predictor than C4 for the response to colesevelam, a bile acid sequestrant medication.^9^ Sequestrants remain the principal treatments for BAD, and guidelines for their use have not notably changed since the 1960s when the disease was first recognized.

Bacteria are essential in transforming primary bile acids into secondary bile acids, and many enzymatic pathways have been described in multiple bacterial species.^10^ The profile of an individual’s bile acid pool is central in maintaining bile acid homeostasis as bile acids regulate their own synthesis. Microbiotas that differ in composition share some degree of functional redundancy, producing similar enzymes or metabolite profiles.^11^ Therefore, to determine the bile acid–transforming potential of the microbiota and to elucidate key bacterial strains potentially responsible for causing BAD, analyses of the functional genes is imperative in studies of the microbiome. Several studies have used 16S rRNA to compare microbial taxa found in BAD patients diagnosed using SeHCAT,^12^^,^^13^ or in studies in BAD diagnosed with other methods.^14^^,^^15^ To date, there has been only one metagenomic functional analysis of the microbiome in irritable bowel syndrome (IBS) cohorts, which included some bile acid malabsorption patients (ie, PBAD) diagnosed by SeHCAT.^16^

Recently, Baumgartner and colleagues reported the presence of biofilms by ileocolonoscopy, associated with excessive levels of fecal bile acids in 57% of IBS-D and mixed IBS patients.^17^ In the absence of SeHCAT scanning, IBS-D with excessive amounts of fecal bile acids is often diagnosed as BAD.^14^^,^^18^ Biofilms could affect ileal bile acid reabsorption by acting as a physical barrier and also alter bile acid-mediated feedback regulation. We hypothesized that such changes could provide a pathophysiologic basis for PBAD.

The aim of this study was to use shotgun metagenomics to analyze and compare specific genes of the microbiome, including those involved in biofilms and bile acid transformation, together with extended bile acid composition profiles in stool samples from healthy donors and BAD patients, as defined by SeHCAT retention. By identifying the associated bacterial species and genes, a greater understanding of the mechanisms that lead to the development of PBAD should be gained to improve the diagnostic and therapeutic options available for those afflicted with this condition.

## Materials and Methods

### Subject Recruitment and Sample Collection

Patients with BAD were recruited for this cross-sectional case-control study. The diagnosis of BAD was made by SeHCAT testing, with a retention value of ≤15%, and a history of chronic diarrhea lasting longer than 3 months. In the total group of 26 patients, 5 had undergone cholecystectomy, 2 were on metformin, and 1 had a diagnosis of Crohn’s disease with no current activity and did not have a SeHCAT test. The M:F ratio was 10:16, the median age was 51.0 (interquartile range (IQR) 47.5–59.2), and the median body mass index was 27.8 (IQR 25.7–33.1). BAD samples were collected from November 2014 to November 2019 at the University Hospital Coventry & Warwickshire. Ethical approval for this study was obtained from the Warwickshire Research Ethics Committee (09/h1211/38). The study size was determined based on the availability of patients with confirmed BAD diagnosed by SeHCAT.

A control group (n = 21) was comprised of healthy adult volunteers who met the following criteria: no diagnosis of gut-related diseases such as IBS, coeliac disease, or inflammatory bowel disease (IBD) within the past year, and normal bowel movements. The M:F ratio was 2:1, the median age was 34.0 (IQR 28–52), and the median body mass index was 25.9 (IQR 23.2–27.6). They had no significant medical history, were omnivorous, nonsmokers, on no medication, and not pregnant. Control samples were collected from April 2017 to May 2018 at University Hospitals Plymouth. The study received approval from the NHS Health Research Authority (18/NW/0346). Written informed consent was obtained from all participants. Individuals who had taken gut microbiota-altering agents, specifically probiotics or antibiotics, within the last 3 months were excluded from the study. Stool samples were collected and stored at −80 °C before processing. Before aliquoting and subsequent extractions, the fecal samples were thawed and thoroughly mixed to ensure homogeneity.

### DNA Extraction and Metagenomic Analysis

DNA was extracted from 0.2 g of stool using the DNeasy PowerSoil Kit (Qiagen) to facilitate downstream microbiota and functional metagenomic analysis, following the manufacturer's protocol. For library preparation, 5 ng/μL of DNA was used with the Illumina DNA Prep kit (Illumina). Sequencing was conducted on a NextSeq 500 platform (Illumina) with the NextSeq 500/550 v2.5 Kits (Illumina), producing paired-end 150 bp reads. Each sample was sequenced to a depth of approximately 50 million reads. Postsequencing, adapter trimming, quality trimming, quality filtering, and length filtering were performed using BBDuk, while host contamination was filtered out using BBMap with the GRCh38 version of the human genome. Both BBDuk and BBMap are part of BBTools suite (version 37.62).^19^ Full DNA sequence quality control parameters can be found in the Supplementary Methods.

The microbial community composition was profiled at the species level using MetaPhlAn version 3.1, leveraging metagenomic shotgun sequencing data rather than traditional 16S rRNA gene sequencing.^20^ The R-based phyloseq package was used for importing, storing, and analyzing phylogenetic sequence data generated by MetaPhlAn. Functional profiling of microbial genes was carried out using the HUMAnN version 3.0.3 pipeline to determine the abundance of microbial functional genes within the metagenomic sequencing data.^20^^,^^21^ Detailed analysis functional genes can be found in Supplementary Methods. Specifically, the analysis targeted genes involved in the conversion of primary to secondary bile acids, aiming to assess the potential for secondary bile acid synthesis, such as bile salt hydrolase (*bsh*) genes responsible for bile acid deconjugation. Additionally, genes within the bile acid-inducible operon (*baiA*, *baiB*, *baiCD*, *baiE*, *baiF*, *baiN*) and hydroxysteroid dehydrogenase (*hdhA*) genes, which are involved in bile acid dehydrogenation and dehydroxylation, as well as genes related to bacterial bile acid transport, were examined. Functional genes involved in biofilm formation were analyzed, including the gene encoding the biofilm regulator protein BssS (*bssS*), as well as those responsible for the synthesis of poly-β-1,6-N-acetyl-D-glucosamine (PGA), such as *pgaA*, *pgaB*, *pgaC*, and *pgaD*.

### Bile Acid Composition Analysis

See Supplementary Methods for additional methodological details and procedural steps. In brief, bile acids were extracted from stool using a methanol extraction method adapted from Lin et al.^22^ Semiquantitative results were obtained for 24 bile acids using calibration curves of the corresponding authentic reference material. Details of the semiquantified compounds, including internal standards, lower and upper limits of quantification are shown in Table 1. Relative abundance (area under the chromatographic peak) results were obtained for 14 additional bile acids (Table 1), which were annotated using reference chemical standards analyzed at the beginning and end of the fecal samples run. The analysis was performed using ultra-high-performance liquid chromatography—high-resolution mass spectrometry, following the protocol established by Sarafian et al.^23^

**Table 1:** Measured BAs

Bile acid name	Abbreviation	Classification	Linear range (micromole/L)		IS
			LLOQ	ULOQ	
Semiquantified with own labeled analog					
Cholic acid	CA	Primary	0.25	500	CA-d4
Chenodeoxycholic acid	CDCA	Primary	0.25	500	CDCA-d4
Glycocholic acid	GCA	Primary, glycine conjugation	0.04	10	GCA-d4
Glycochenodeoxycholic acid	GCDCA	Primary, glycine conjugation	0.04	10	GCDCA-d4
Taurocholic acid	TCA	Primary, taurine conjugation	0.04	10	TCA-d4
Taurochenodeoxycholic acid	TCDCA	Primary, taurine conjugation	0.01	10	TCDCA-d9
Deoxycholic acid	DCA	Secondary	0.25	500	DCA-d4
Lithocholic acid	LCA	Secondary	0.05	100	LCA-d4
Glycodeoxycholic acid	GDCA	Secondary, glycine conjugation	0.04	10	GDCA-d4
Taurodeoxycholic acid	TDCA	Secondary, taurine conjugation	0.005	10	TDCA-d4
Glycolithocholic acid	GLCA	Secondary, glycine conjugation	0.04	10	GLCA-d4
Taurolithocholic acid	TLCA	Secondary, taurine conjugation	0.04	10	TLCA-d4
Glycoursodeoxycholic acid	GUDCA	Secondary, glycine conjugation	0.005	10	GUDCA-d4
Tauroursodeoxycholic acid	TUDCA	Secondary, taurine conjugation	0.04	10	TUDCA-d4
Semiquantified with alternative labeled analog					
Ursodeoxycholic acid	UDCA	Secondary	0.1	100	HDCA-d4
Isolithocholic acid	Iso-LCA	Iso-BA	0.005	10	LCA-d4
Semiquantified without an internal standard					
Isocholic acid	Iso-CA	Iso-BA	0.0125	25	None
Isochenodeoxycholic acid	Iso-CDCA	Iso-BA	0.1	100	None
Isodeoxycholic acid	Iso-DCA	Iso-BA	0.0125	25	None
Isoursodeoxycholic acid	Iso-UDCA	Iso-BA	0.025	25	None
7-oxodeoxycholic acid	7-oxo-DCA	Oxo (keto)-BA	0.1	100	None
7-oxolithocholic acid	7-oxo-LCA	Oxo (keto)-BA	0.05	100	None
12-oxolithocholic acid	12-oxo-LCA	Oxo (keto)-BA	0.1	100	None
ω-muricholic acid	ω-MCA	Muricholic acid	0.2	8	None
Relative abundance					
Cholic acid-3-sulfate	CA-3S	Primary, sulfated	n/a		
Chenodeoxycholic acid-3-sulfate	CDCA-3S	Primary, sulfated	n/a		
Deoxycholic acid-3-sulfate	DCA-3S	Secondary, sulfated	n/a		
Lithocholic acid-3-sulfate	LCA-3S	Secondary, sulfated	n/a		
Ursodeoxycholic acid-3-sulfate	UDCA-3S	Secondary, sulfated	n/a		
5α-cholanic acid-3-one	5α-CA-3-one	Allo-BA	n/a		
5β-cholenic acid-7α-ol-3-one	5β-CA-7α-ol-3-one	Oxo (keto)-BA	n/a		
5β-cholanic acid 12α-ol-3-one	5β-CA-12α-ol-3-one	Oxo (keto)-BA	n/a		
Isoallolithocholic acid	Iso-allo-LCA	Iso-allo-BA	n/a		
Allolithocholic acid	Allo-LCA	Allo-BA	n/a		
3-oxo-cholanic acid	3-oxo-CA	Oxo (keto)-BA	n/a		
3,12-di-oxo-cholanic acid |3,6-di-oxo-cholanic acid	3,12-Di-oxo-CA|3,6-D-oxo-CA	Oxo (keto)-BA	n/a		
3α-hydroxy-7,12-di-oxo-cholanic acid	3α-OH-7,12-Di-oxo-CA	Oxo (keto)-BA	n/a		
3-oxo-cholic acid	3-oxo-CA	Oxo (keto)-BA	n/a		

BA, bile acid; LLOQ, lower limit of quantification; ULOQ, upper limit of quantification.

### Statistical Analysis

Data analysis and graph generation were performed using Prism 9 (GraphPad Software, San Diego, California, USA) and packages within the R environment.^24^ The Shapiro–Wilk normality test indicated that the HUMAnN v3.0.3 and MetaPhlAn v3.1 normalized reads, along with bile acid measurements, were nonparametrically distributed. Consequently, nonparametric statistical methods were employed.

Comparative analyses were conducted using the Mann–Whitney U test for unpaired data, with results expressed as medians accompanied by 95% confidence intervals unless otherwise specified. Alpha diversity of the gut microbiome was assessed via the Mann–Whitney U test, with *P* values adjusted using the Bonferroni correction. Beta diversity was evaluated using the Bray-Curtis dissimilarity index and visualized through nonmetric multidimensional scaling ordination plots. Correlation analyses used nonparametric methods (Spearman’s rho).

A *P* value of ≤ .05 was considered statistically significant. Significance levels are denoted as follows: ∗*P* ≤ .05, ∗∗*P* ≤ .01, ∗∗∗*P* ≤ .001, ∗∗∗∗*P* ≤ .0001.

## Results

### Taxonomic Analysis

**Figure 1:**
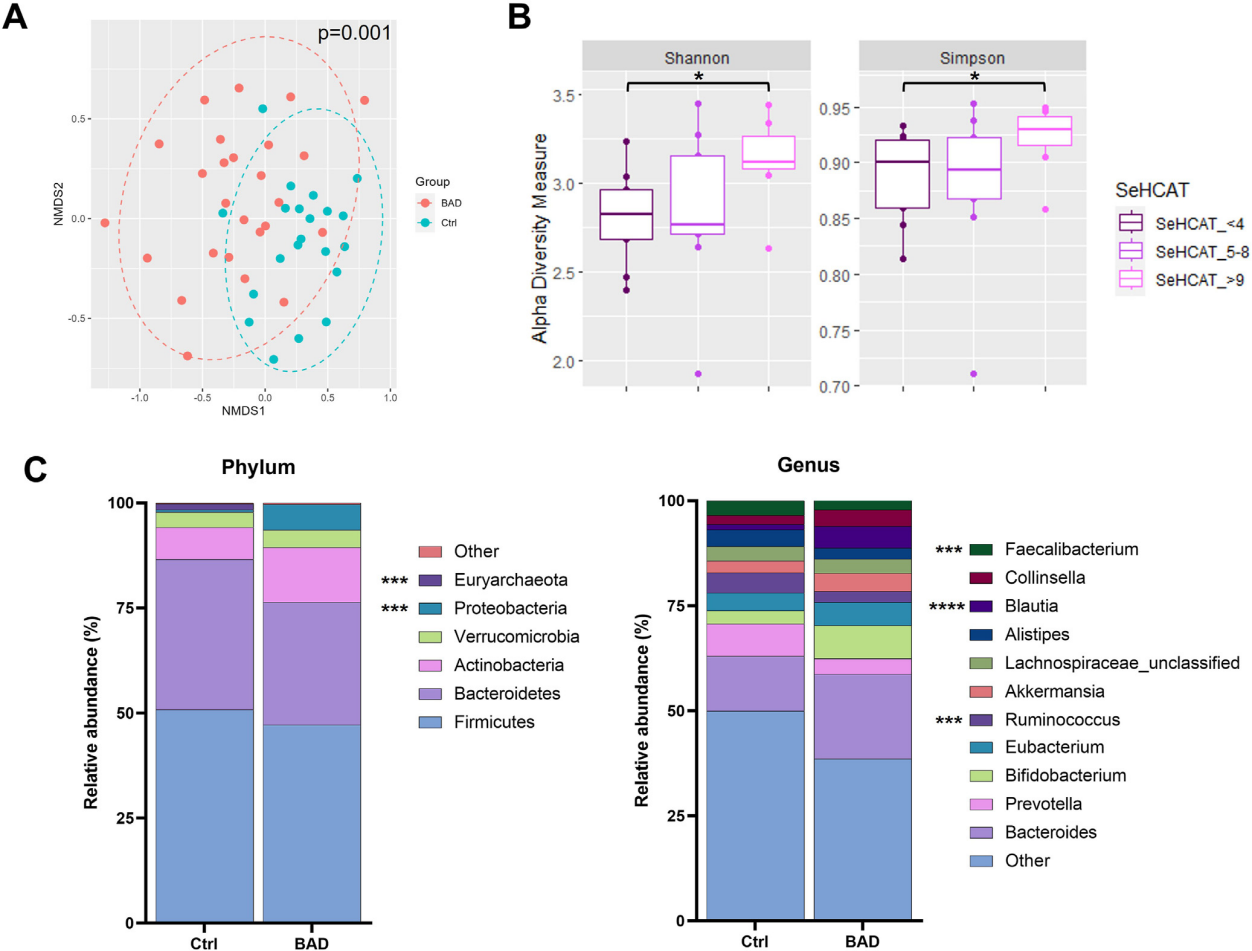
Taxonomic analysis. (A) Beta-diversity visualized using NMDS plot with Bray-Curtis dissimilarity distances. Plot ellipses represent the 95% confidence regions for group clusters. Microbial species clustering by sample groups (*P* ≤ .001). (B) Species richness in BAD patients separated into 3 groups by SeHCAT scores from most (≤4%) to least (≥9%) severe. Box plots depicting Shannon and Simpson alpha diversity indices of each sample: SeHCAT ≤ 4% (dark purple) n = 7, SeHCAT 5%–8% (purple) n = 9, SeHCAT ≥ 9% (light purple) n = 9. Bars representing the medians and IQR. ∗*P* = .031 and *P* = .041 Shannon and Simpson index respectively. (C) Difference in microbial composition at phylum and genus level in BAD and Ctrl samples ∗∗∗*P* ≤ .005, ∗∗∗∗*P* ≤ .0001. Number of samples: Ctrl = 21, BAD = 26 with SeHCAT results in 25. Ctrl, healthy control; NMDS, nonmetric multidimensional scaling.

**Figure A1:**
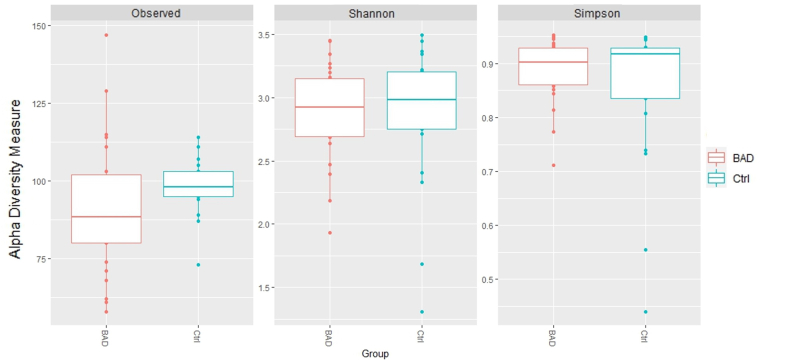
Species richness across sample types BAD (n = 26) and Ctrl (n = 21). Box plots depicting Observed, Shannon and Simpson alpha diversity indices of each sample. Error bars representing the medians and IQR. No significant differences observed (*P* > .05). BAD, bile acid diarrhea; Ctrl, healthy control.

**Figure A2:**
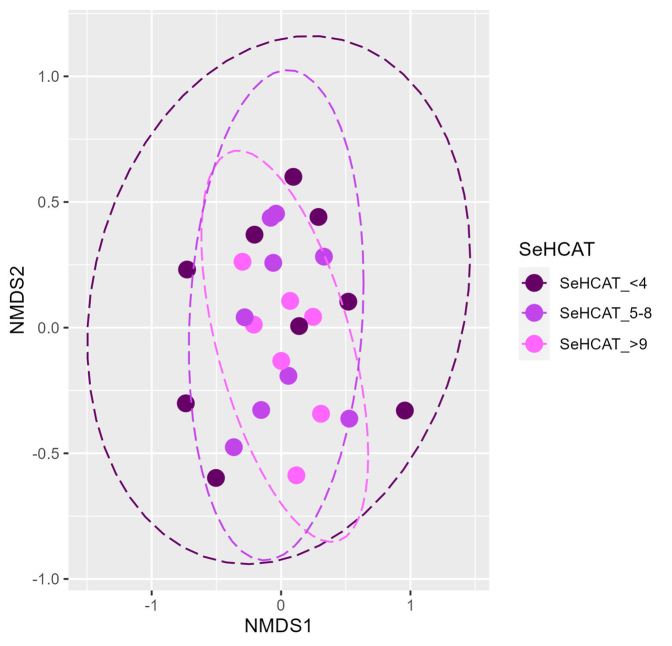
Figure of beta-diversity visualized using NMDS plot with Bray-Curtis dissimilarity distances. Plot ellipses represent the 95% confidence regions for group clusters. Bacterial species clustering by sample groups. Number of samples: SeHCAT ≤ 4% (dark purple) n = 7, SeHCAT 5%–8% (purple) n = 9 and SeHCAT ≥ 9% (light purple) n = 9. No significant differences observed. NMDS, nonmetric multidimensional scaling.

Metagenomic sequence analysis detected 420 different microbial taxa overall. Beta diversity analysis revealed a significant disparity in microbial composition between the BAD and healthy control samples (Figure 1A). Conversely, alpha diversity analysis indicated no significant difference in microbial richness between the 2 groups (Figure A1). To further investigate, BAD samples were stratified based on their SeHCAT retention scores. Three groups of similar size were ordered from most to least severe (Figure 1B). Notably, there was a significant difference in alpha diversity between BAD samples with low retention scores (≤4%, severe BAD) and those with high SeHCAT retention scores (≥9%, mild BAD), as measured by the Shannon and Simpson index. However, beta diversity analysis within these subgroups did not reveal significant differences (Figure A2). Distinct differences in microbial composition were observed at both the phylum and genus levels between patients with BAD and healthy individuals (Figure 1C).

### Species Comparative Analysis

Several microbial species exhibited notable and highly significant differences in their frequency of detection in BAD patients compared to healthy controls (Table 2). *Ruminococcus gnavus* (*R. gnavus*), now classified as *Mediterraneibacter gnavus*,^25^ was detected in over 88% of BAD samples, but in less than 10% of healthy donor samples (Table 2). This difference was significant (*P* ≤ .0001) with an odds ratio of 72.8. *Clostridium innocuum*, *Blautia* sp. CAG 257, *Erysipelatoclostridium ramosum*, *Eggerthella lenta,* and *Escherichia coli* (*E. coli*) were also much more likely to be found in BAD patients. In contrast, *Firmicutes bacterium CAG 110*, *Eubacterium siraeum*, *Oscillibacter* sp. 57_20, and *Oscillibacter* sp. CAG 241 were much more likely to be detected in the control group, with odds ratios ranging from 25 to 30 (Table 2). Odds ratios for other notable species are also shown, with reduced likelihood of detection in BAD of *Ruminococcus bicirculans*, *Ruminococcus bromii,* and *Alistipes shahii*. Interestingly, the pattern of species defined by high *R. gnavus* and low *Oscillibacter* spp. present in most of the PBAD patients, was also found in 4 of the 5 patients with cholecystectomy and the Crohn’s patient, but not in the 2 BAD patients who were on metformin.

**Table 2:** Bacterial Species Detected More Frequently in Individuals With BAD and Control

Bacterial species detected more often in individuals with BAD							
Species	% Of group with species				95% CI	*P*	SeHCAT correlation
	% BAD	% Ctrl	Diff %	OR			
*Ruminococcus gnavus*	88	10	79	72.8	(11.0–481.9)	.0001	−0.53, 0.006^a^
*Clostridium innocuum*	69	14	55	13.5	(3.1–59.2)	.0006	−0.10, 0.635
*Blautia sp CAG 257*	58	10	48	13.0	(2.5–67.6)	.0024	−0.33, 0.109
*Erysipelatoclostridium ramosum*	65	19	46	13.0	(2.5–67.6)	.0024	−0.31, 0.134
*Eggerthella lenta*	85	43	42	7.3	(1.9–28.9)	.0044	−0.12, 0.560
*Escherichia coli*	81	48	33	4.6	(1.3–16.9)	.0208	0.20, 0.343
*Clostridium leptum*	54	38	16	1.9	(0.6–6.1)	.2842	0.08, 0.695

Bacterial species detected more often in control individuals							
Species	% BAD	% Ctrl	Diff %	OR	95% CI	*P*	SeHCAT correlation
*Firmicutes bacterium CAG 110*	8	71	−64	30.0	(5.3–168.5)	.0001	0.38, 0.064
*Eubacterium siraeum*	19	86	−66	25.2	(5.3–120.4)	.0001	0.41, 0.041^b^
*Oscillibacter sp 57 20*	19	86	−66	25.2	(5.3–120.4)	.0001	0.33, 0.102
*Oscillibacter sp CAG 241*	12	76	−65	24.5	(5.1–117.6)	.0001	0.35, 0.087
*Ruminococcus bicirculans*	27	81	−54	11.5	(2.9–46.4)	.0006	0.36, 0.073
*Ruminococcus_bromii*	35	81	−46	8.0	(2.1–31.2)	.0026	0.22, 0.12
*Alistipes shahii*	31	76	−45	7.2	(2.0–26.5)	.0030	0.20, 0.328

The percentages of individuals with identified species in the BAD (n = 26) and control (Ctrl; n = 21) groups are shown, with the percentage difference between these (Diff %). In (a), species found in >66% of BAD, and <33% of controls, and some selected others are shown. In (b) species found in >66% of controls and <36% of BAD, individuals are shown. Identified species had >1000 reads per individual (mean total reads = 50 million). The odds ratios (OR) for the percentages, 95% confidence intervals (95% CI), and *P* value were calculated. Spearman correlation analyses between the relative abundance of SeHCAT, values and the species are also shown, together with their *P* values.

OR, odds ratio.

a *P* > .01.

b *P* > .05.

**Figure A3:**
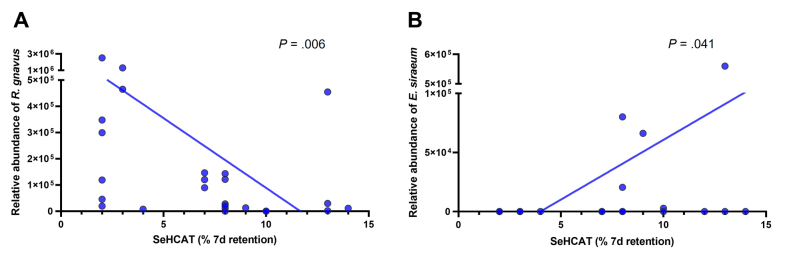
Correlation analysis between the abundance of (A) *R. gnavus* (rho = −0.53, *P* = .006) and (B) *Eubacterium siraeum* (*E. siraeum*) (rho = 0.41, *P* = .041) with SeHCAT values, determined using Spearman's correlation. The simple linear regression line is also shown. Abundance is shown as the number of identified reads per individual (n = 25).

There was a significant negative correlation between the relative abundance of *R. gnavus* and SeHCAT retention values (Spearman Rho −0.53, *P* = .008), (Figure A3). Conversely, *E. siraeum* demonstrated a positive correlation with SeHCAT values (rho = 0.41, *P* = .041). No significant correlation was observed between SeHCAT and the other bacteria listed in Table 2.

**Figure A4:**
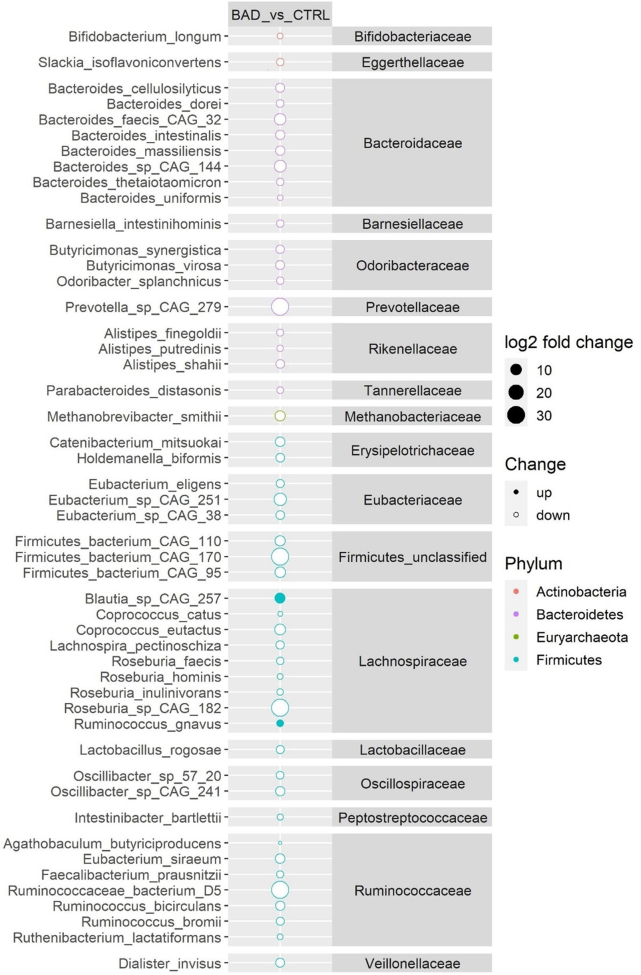
A total of 49 species with high base mean abundance: Log2 fold differences in mean values (as determined with DESeq2) between bacterial species in healthy control samples and bile acid diarrhea are illustrated. Bacterial taxonomies: genera and species are shown on the left while family and group are on the right. Only significant findings (*P* ≤ .05 after correction for multiple comparisons) are shown. Size of dots signify log2 fold-change, filled dots represent higher species abundance, empty dots represent lower species abundance in BAD samples. Dots are colored based on bacterial phylum. Number of samples: Ctrl = 21, BAD = 26.

**Figure A5:**
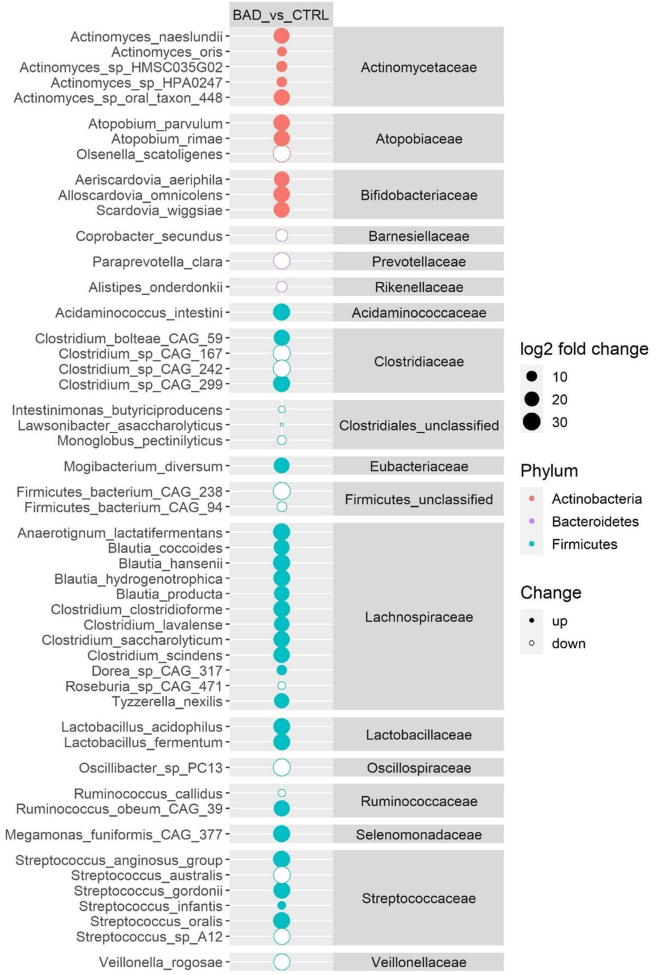
50 species with lower base mean abundance: Log2 fold differences in mean values (as determined with DESeq2) between bacterial species in healthy control samples and bile acid diarrhea are illustrated. Bacterial taxonomies: genera and species are shown on the left while family and group are on the right. Only significant findings (*P* ≤ .05 after correction for multiple comparisons) are shown. Size of dots signify log2 fold-change, filled dots represent higher species abundance, empty dots represent lower species abundance in BAD samples. Dots are colored based on bacterial phylum. Number of samples: Ctrl = 21, BAD = 26.

When the mean abundance of bacterial species in the BAD and healthy control groups was compared, 99 species showed significant differences. Of the 49 most abundant species found in healthy volunteers, only *Blautia* sp CAG 257 and *R. gnavus* were higher in BAD. *Firmicutes bacterium* CAG 170, *Prevotella* sp CAG 279, *Roseburia* sp CAG 182, and *Ruminococcaceae bacterium* D5 all had 30-fold lower mean values in BAD patients (Figure A4). Key bacterial species associated with a healthy microbiome, *Bifidobacterium longum* (*B. longum*) and *Faecalibacterium prausnitzii* (*F. prausnitzi*) had a lower presence in BAD patients. In contrast, many species that were less abundant in healthy volunteers had higher relative mean values in BAD patients, and only 17 of 50 species showed lower abundance (Figure A5).

### Analysis of Bile Acid–Transforming Genes

**Figure 2:**
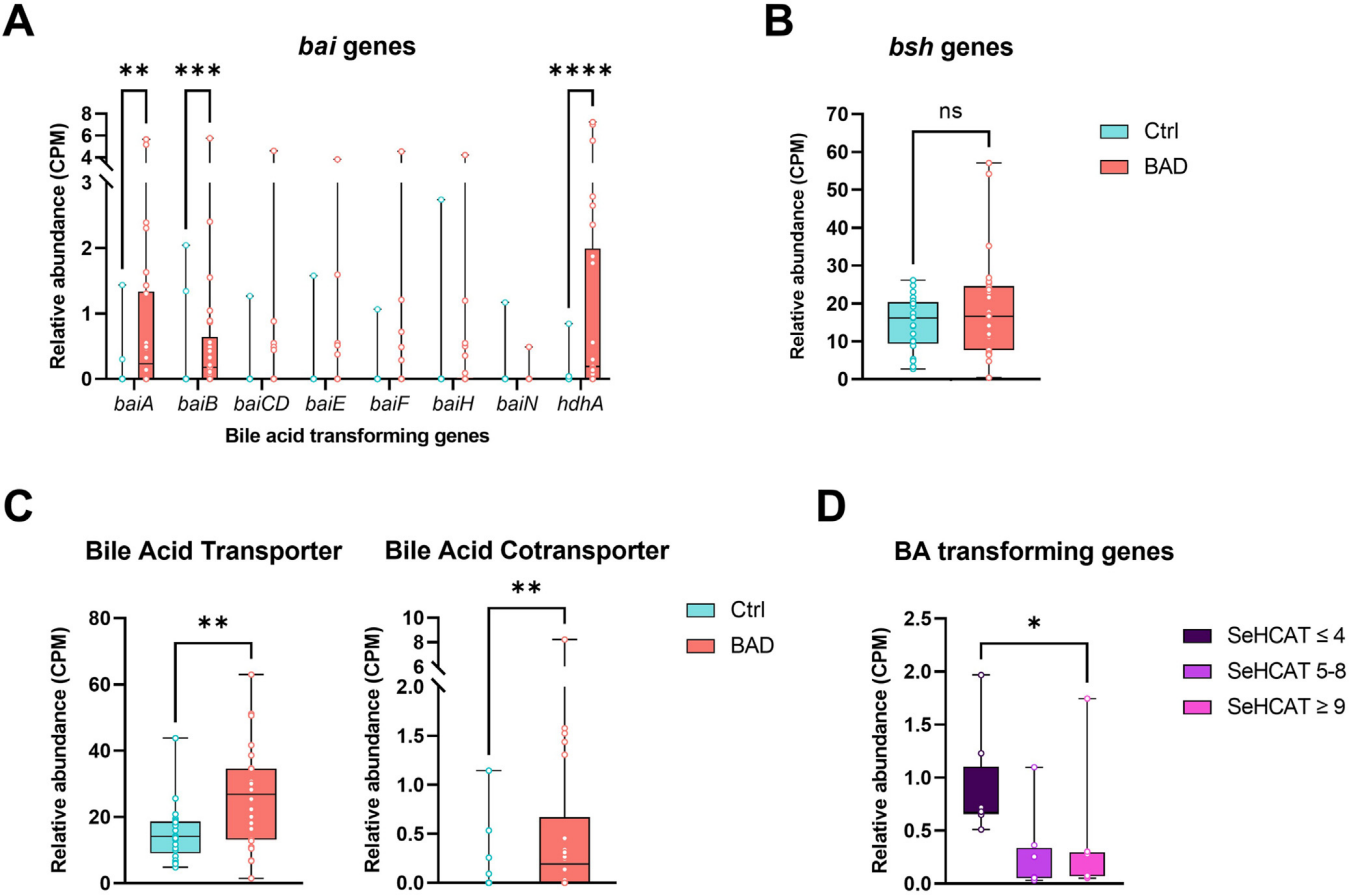
Relative abundances of bile acid–transforming genes. (A) *baiA, baiB, baiCD, baiE, baiF, baiH, baiN,* and *hdhA* (B) *bsh* (C) Bile acid transporter and bile acid cotransporter genes. Number of samples: Ctrl = 21, BAD = 26. (D) Relative abundances of bile acid–transforming genes in bile acid diarrhea patients separated by SeHCAT scores from most (≤4) to least (≥9) severe. The following genes *bsh, baiA, baiB, baiCD, baiE, baiF, baiH, baiN* and *hdhA*. SeHCAT ≤ 4 (dark purple) n = 7, SeHCAT 5–8 (purple) n = 9, SeHCAT ≥ 9 (light purple) n = 9. The box-and-whisker plots show the medians (horizontal lines), IQR (boxes), and maximum and minimum points (whiskers). Differences were assessed with the Mann–Whitney test. ∗*P* ≤ .05, ∗∗*P* ≤ .01, ∗∗∗*P* ≤ .005, ∗∗∗∗*P* ≤ .0001. A, B, C, D, E, F, H and N, *baiA, baiB, baiCD, baiE, baiF, baiH, baiN*; BA, bile acid; BAI, bile acid-inducible; BSH, bile salt hydrolase; CPM, copies per million; Ctrl, healthy control; HDHA, hydroxysteroid dehydrogenase.

The relative abundance of bile acid-transforming genes *baiA*, *baiB*, and *hdhA* was significantly higher in stool samples from BAD patients compared to healthy controls (Figure 2A). No significant differences were found for *baiCD*, *baiE*, *baiF*, *baiH*, *baiN*, or *bsh* genes (Figure 2A and B). However, microbial bile acid transporter and cotransporter genes (Kegg Ortholog K03453 and K03453, respectively) were significantly more abundant in BAD patients (Figure 2C). A combination of all bile acid genes showed a clear difference in relative abundance between severe and mild forms of BAD stratified by SeHCAT scores (Figure 2D).

### Fecal Bile Acid Composition

**Figure A6:**
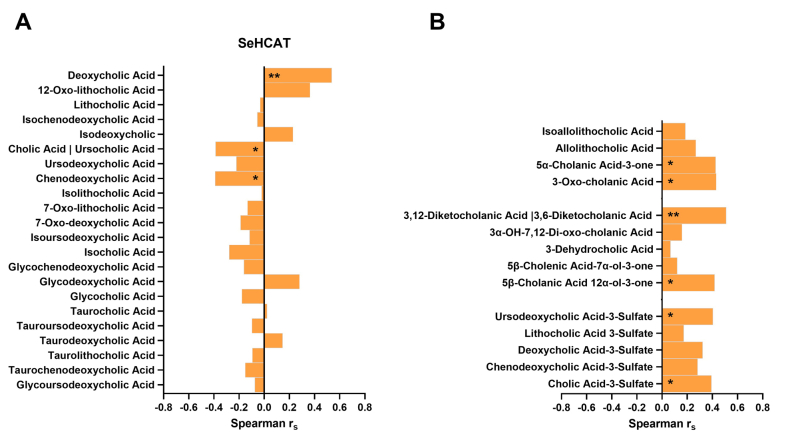
Spearman's correlation analysis between the SeHCAT score and (A) semiquantification and (B) relative abundance of fecal bile acids. ∗*P* ≤ .05, ∗∗*P* ≤ .01, ∗∗∗*P* ≤ .005, ∗∗∗∗*P* ≤ .0001.

Bile acid untargeted profiling and targeted methods have been reported previously.^23^ In this work, for the first time elements of both approaches have been combined and run on a high-resolution time-of-flight mass spectrometer. In comparison to the healthy control group, the BAD group exhibited a 3.27-fold higher median value of total quantified fecal bile acid levels, with concentrations of individual bile acids significantly elevated (Table 3). As previously reported, the majority of bile acids were unconjugated, with low levels of glyco- and tauro-conjugated forms. Primary bile acids constituted 7% of total bile acids in controls and 15% in BAD when iso-variants were included. DCA was the most abundant bile acid in both groups (50% in controls, 60% in BAD), followed by LCA, which accounted for 25% in controls but only 10% in BAD. Notably, 12-oxo-LCA acid was the second most common bile acid in BAD, present at 11% in both groups. Iso-forms were generally more prevalent in BAD, with Iso-UDCA acid showing an 8-fold higher compared to healthy controls, alongside a marked rise in the intermediate 7-oxo-LCA. For sulfated bile acids (Table 4), UDCA-3S exhibited the largest increase, with a median ratio of 8.9 between BAD and control groups. Interestingly, bile acids involved in the allo-pathway (3-oxo-CA, 5α-CA-3-one, Allo-LCA, and Iso-allo-LCA) were less abundant in the BAD group. In particular, Iso-allo-LCA acid showed a striking decrease, with a median BAD-to-control ratio of just 0.1 (*P* < .0001). Additionally, SeHCAT retention scores were negatively correlated with primary bile acids CA|UCA and CDCA, while a positive correlation was observed with secondary bile acids, including DCA, 5α-CA-3-one, 3-oxo-CA, and UDCA-3S (Figure A6). Note, CA and UCA, could not be chromatographically separated and the reported amount may contain both compounds.

**Table 3:** Semiquantification of Fecal BAs

Semi-quantification (Micromole/L in extract)^a^	Control			BAD			Median ratio	*P*
	Median	IQR	% Of total	Median	IQR	% Of total	(BAD/Ctrl)	
Total quantified BA	102.71	(64.86–166.93)		335.80	(250.46–518.74)		3.27	<.0001
Deoxycholic acid	45.28	(16.67–86.98)	50.22	155.73	(76.07–194.53)	60.08	3.44	.0004
12-oxo-lithocholic acid	10.04	(4.59–24.66)	11.14	28.38	(8.38–43.02)	10.95	2.83	.1031
Lithocholic acid	22.44	(8.57–27.19)	24.89	26.60	(13.51–39.15)	10.26	1.19	.1868
Isochenodeoxycholic acid	3.62	(1.66–7.69)	4.01	15.46	(5.96–23.01)	5.97	4.28	.0008
Isodeoxycholic	2.12	(1.17–5.23)	2.35	8.96	(4.80–12.27)	3.46	4.22	.0003
Cholic acid |Ursocholic acid^b^	1.04	(0.37–1.27)	1.15	6.76	(1.91–52.76)	2.61	6.52	.0099
Ursodeoxycholic acid	1.56	(0.60–2.59)	1.73	5.20	(3.05–21.59)	2.00	3.33	<.0001
Chenodeoxycholic acid	0.89	(0.29–1.43)	0.99	4.87	(1.44–25.22)	1.88	5.45	<.0001
Isolithocholic acid	1.71	(0.69–3.14)	1.90	1.84	(0.68–3.73)	0.71	1.08	.6965
7-Oxo-lithocholic acid	0.20	(0.11–0.65)	0.22	1.55	(0.42–7.88)	0.60	7.69	.0011
7-Oxo-deoxycholic acid	0.29	(0.19–1.31)	0.32	1.06	(0.50–8.59)	0.41	3.71	.0069
Isoursodeoxycholic acid	0.11	(0.04–0.21)	0.12	0.91	(0.31–3.05)	0.35	8.32	.0006
Isocholic acid	0.05	(0.02–0.12)	0.06	0.72	(0.38–3.32)	0.28	14.08	.0007
Sum of specific classes								
Conjugates (Glyco + Tauro)	0.32	(0.30–0.34)	0.35	0.61	(0.42–1.82)	0.24	1.92	<.0001
All primaries	1.83	(0.78–3.18)	2.18	9.75	(1.59–127.25)	7.28	5.32	.0088
Iso-BA (3β-OH)	6.34	(3.92–13.12)	7.50	29.75	(13.47–53.81)	8.43	4.69	<.0001
All primaries + iso-forms	8.54	(4.05–11.92)	6.58	39.09	(18.57–151.12)	15.06	4.58	.0002
Oxo-BA	10.84	(5.12–37.60)	12.30	42.53	(18.08–69.18)	12.94	3.93	.0060

BA, bile acid.

a Bile acids were semiquantified as micromole/L (mM) in the extracts. Rows are arranged in descending order of median abundance in the BAD, group. Glycochenodeoxycholic, glycocholic, glycodeoxycholic, glycolithocholic, glycoursodeoxycholic, taurochenodeoxycholic, Taurocholic, taurodeoxycholic, taurolithocholic, and tauroursodeoxycholic and omega-muricholic acid were detected at low levels (<1 mM in all subjects) or were below the lower limit of quantification and are not individually shown or included in the calculation of the % of total.

b Cholic acid and ursocholic acid, with identical molecular formula, could not be chromatographically separated and the reported amount may contain both compounds.

**Table 4:** Relative Abundance of Fecal BAs

Relative abundance	Control			BAD	Median ratio	*P*
	Median	IQR	Median	IQR	(BAD/Ctrl)	
Cholic acid-3-sulfate	1.85	(1.50–3.43)	1.97	(1.30–13.27)	1.06	.8259
Chenodeoxycholic acid-3-sulfate	2.65	(1.91–13.10)	7.81	(3.16–162.44)	2.95	.0251
Deoxycholic acid-3-sulfate	3.33	(2.54–26.60)	4.45	(2.73–38.61)	1.33	.4654
Lithocholic acid 3-sulfate	2.17	(1.56–8.54)	10.39	(2.42–66.43)	4.79	.0168
Ursodeoxycholic acid-3-sulfate	0.89	(0.58–5.33)	7.94	(1.86–126.83)	8.93	.0012
5β-Cholanic acid 12α-ol-3-one	391.89	(189.85–766.86)	1258.14	(369.65–2362.79)	3.21	.0135
5β-Cholenic acid-7α-ol-3-one	4.69	(2.55–16.76)	15.88	(4.74–97.93)	3.39	.0160
3-Dehydrocholic acid	2.47	(1.93–4.91)	17.15	(5.19–85.12)	6.93	.0004
3α-OH-7,12-Di-oxo-cholanic acid	1.21	(0.70–2.76)	3.10	(1.31–19.64)	2.55	.0091
3,12-Di-oxo-cholanic acid^a^	283.35	(120.78–1368.14)	360.33	(51.94–873.08)	1.27	.5823
3-Oxo-cholanic acid	720.54	(214.15–1090.58)	344.62	(106.74–1023.98)	0.48	.2713
5α-Cholanic acid-3-one	7.57	(2.60–2.80)	2.63	(0.89–5.20)	0.35	.0099
Allolithocholic acid	3.48	(1.60–9.14)	0.75	(0.33–2.60)	0.21	.0019
Isoallolithocholic acid	43.33	(15.61–118.97)	4.16	(2.28–12.34)	0.10	.0001
Sums of specific classes						
Sulfates	11.71	(8.73–103.05)	45.21	(17.79–638.61)	3.86	.0083
3-oxo	1797	(7900–3784)	2415	(967–4019)	1.34	.4593
7-oxo	1.21	(0.70–2.76)	3.10	(1.31–19.64)	2.55	.0091
12-oxo	2851	(1378–13,690)	3621	(570–8975)	1.27	.7718
Allo (5α)	57.92	(19.59–180.90)	8.91	(3.51–22.01)	0.15	.0006

BA, bile acid.

a 3,12-Dioxocholanic acid and 3,6-Dioxocholanic and potentially other 6-OH, BAs with identical molecular formula, could not be chromatographically separated and are reported together.

**Figure A7:**
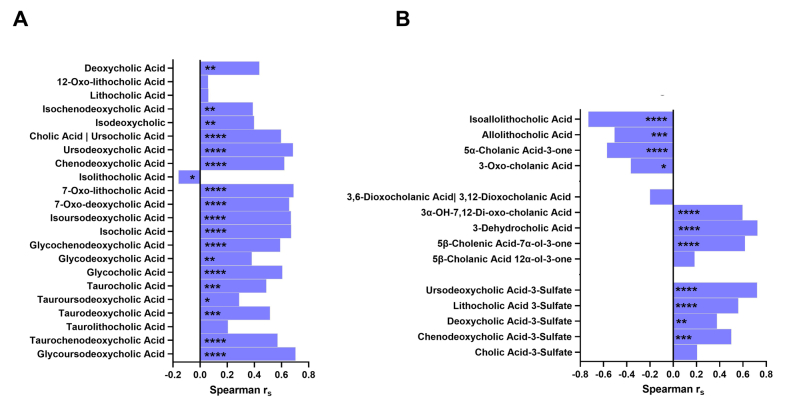
Spearman's correlation analysis between the abundance of *R. gnavus* and (A) semiquantification and (B) relative abundance of specific fecal bile acids. ∗*P* ≤ .05, ∗∗*P* ≤ .01, ∗∗∗*P* ≤ .005, ∗∗∗∗*P* ≤ .0001.

*R. gnavus* also demonstrated a strong positive correlation with the abundance of nearly all primary and secondary bile acids, ordered by their abundance (Figure A7A), including DCA, Iso-CDCA, Iso-DCA, CA|UCA, UDCA, 7-oxo-LCA, 7-oxo-DCA. However, it negatively correlated with Iso-LCA. In the group of bile acids measured with untargeted analysis, *R. gnavus* showed negative correlations with several bile acids including Iso-allo-LCA, Allo-LCA, and 5α-CA-3-one but positive associations with 3α-H-7, 12-Di-oxoCA, 5β-CA-7α-ol-3-one, and most of the 3-sulfated bile acids (Figure A7B). By contrast, no significant correlations were found between *E. coli* or *F. prausnitzii* and bile acids.

### Biofilms

**Figure 3:**
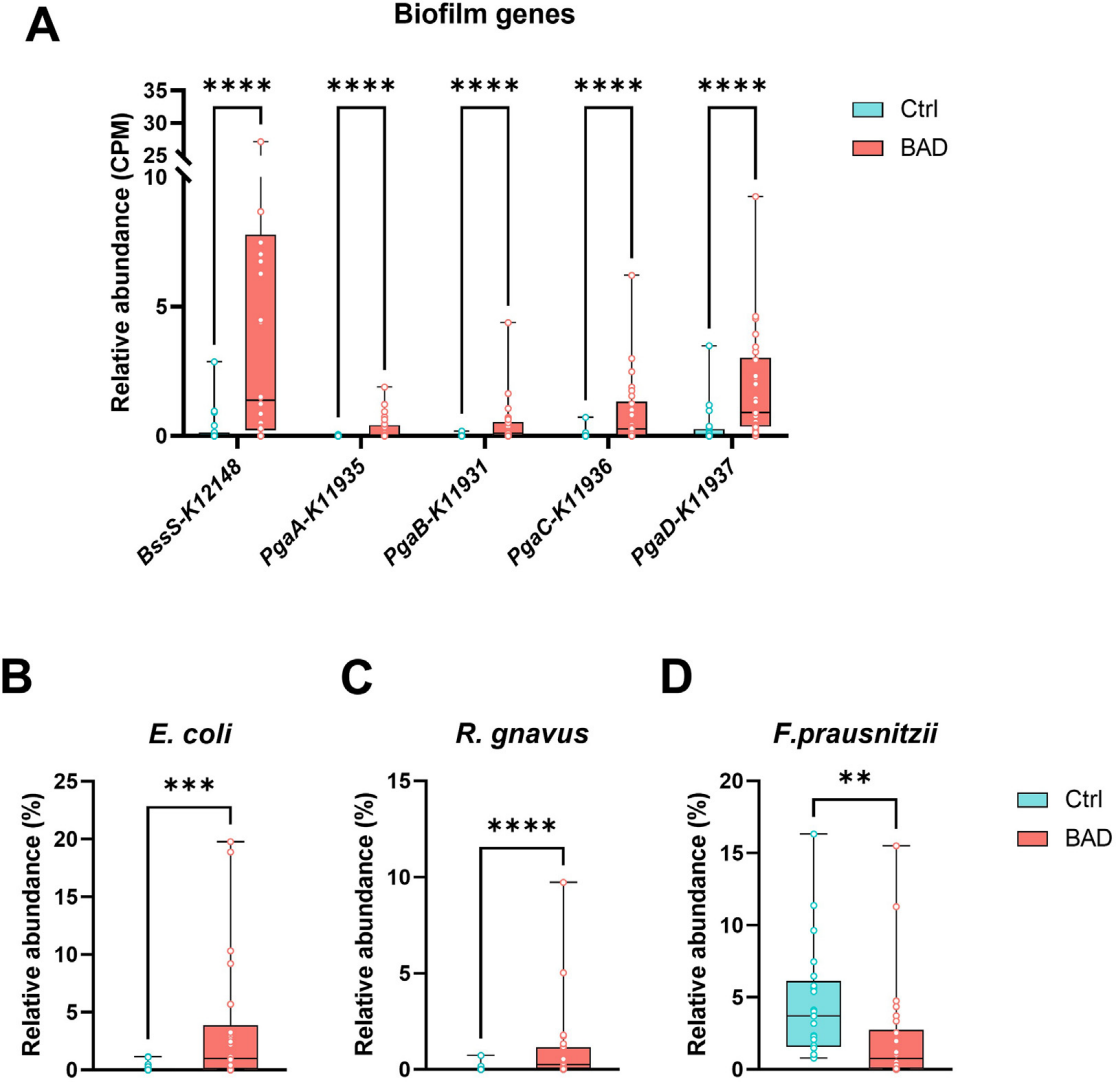
(A) Relative abundance of functional genes associated with biofilm formation. (B) Relative abundance of *E. coli,* (C) *R. gnavus,* and (D) *F. prausnitzii*. The box-and-whisker plots show the medians (horizontal lines), IQR (boxes), and maximum and minimum points (whiskers). Differences were assessed with the Mann–Whitney test. ∗∗*P* ≤ .01, ∗∗∗*P* ≤ .005, ∗∗∗∗*P* ≤ .0001. Number of samples: Ctrl = 21, BAD = 26. bssS, biofilm regulator; Ctrl, healthy control; *E. coli*, *Escherichia coli*; *F. prausnitzii*, *Faecalibacterium prausnitzii*; pgaA, biofilm PGA synthesis protein; pgaB, biofilm PGA synthesis lipoprotein; pgaC, biofilm PGA synthesis N-glycosyltransferase; pgaD, biofilm PGA synthesis protein; *R. gnavus*, *Ruminococcus gnavus*.

The relative median abundance of the biofilm genes (*bssS*, *pgaA*, *pgaB*, *pgaC*, and *pgaD*) was significantly elevated in the BAD group in comparison to the healthy control group (*P* values of ≤ .00008; Figure 3A). Bacterial species *E. coli*, *R. gnavus*, and *F. prausnitzii* have previously been observed to correlate with ileal and colonic biofilms.^17^ In this study, we found a statistically significant greater abundance of *E. coli* and *R. gnavus*, and a significant lower abundance of *F. prausnitzii* in BAD patients (Figure 3B–D).

## Discussion

Our results provide new evidence for the role of *R. gnavus,* biofilm formation, and specific metabolic pathways for the dysregulation of bile acid metabolism in BAD patients diagnosed by SeHCAT.

Metagenomic shotgun sequencing enabled the identification of over 400 different taxa with greater accuracy than 16S rRNA analysis and allowed specific metabolic pathways to be defined in BAD patients. Alpha diversity, reflecting microbial richness, showed no significant differences compared to healthy individuals, whereas beta diversity revealed distinct differences in bacterial composition in BAD patients. This indicates that although the overall number of bacterial species remains similar, the bacterial species present in BAD patients differ notably from those in healthy individuals. This suggests that changes in the composition of the gut microbiota, rather than a reduction in microbial richness, may play a more significant role in the pathophysiology of BAD. These findings align with a previous study that also used metagenomic shotgun sequencing to compare stool samples from patients with IBS-D and high fecal bile acid levels with those from healthy controls.^14^ Within our BAD group, a reduction in alpha diversity was observed as SeHCAT retention values decreased, an indication of more severe disease. However, beta diversity analysis did not show significant changes in microbial composition with varying disease severity. These findings suggest that microbial richness, rather than compositional shifts, may be more closely associated with the progression of BAD, emphasizing the importance of microbial diversity in maintaining gut health. This is consistent with published research categorizing BAD samples by SeHCAT retention, which also reported no significant beta diversity changes between groups. However, in contrast to the current study, the alpha diversity was not reported.^16^

Several microbial species demonstrated significant differences in their detection frequency between BAD patients and healthy controls. These findings suggest a distinct microbial signature associated with BAD, characterized by an overrepresentation of certain species in patients and a notable absence or reduced detection of other species compared to healthy individuals. In particular, we found that *R. gnavus* is a microbial marker for BAD. *R. gnavus* is thought to be widely distributed at low abundance (∼0.1%) in stools from healthy people^26^ and is over-represented in patients with IBD, such as Crohn's disease and ulcerative colitis, IBS, and diabetes.^27^ *R. gnavus* produces an inflammatory polysaccharide, which could affect the barrier function and microbiome composition of the gut.^26^ In addition, *R. gnavus* and several other species are known to have genes capable of modifying bile acids and contributing to biofilm development (see below). Among the most interesting taxa that are reduced in BAD patients are *Oscillibacter s*pp, which have been recently linked to cholesterol metabolism and bile acids.^28^ The study of the large Framingham cohort described how subjects could be assigned to 1 of 2 clusters represented by either *Oscillibacter* or *R. gnavus*. The *R. gnavus* cluster was typically associated with higher cholesterol levels and less efficient conversion of bile acids. These findings provide a compelling comparison to our results and suggest potential avenues for future research.

The negative correlation between the relative abundance of *R. gnavus* and SeHCAT retention supports our hypothesis that *R. gnavus* plays a key role in bile acid dysregulation and indicates a potential mechanistic link between this species and the development and exacerbation of BAD. The positive correlation between *E. siraeum* and improved SeHCAT retention could be relevant for a therapeutic strategy aimed at modulating gut microbiota in BAD patients. More generally, our findings can help to narrow the focus on relevant microbial targets for potential interventions to manage BAD.

Shifts in the microbial composition were observed, with 99 species exhibiting substantial changes in mean abundance between BAD and healthy control groups. These findings consistently suggest a profound microbial imbalance in BAD patients, characterized by overgrowth of specific taxa, including *Blautia* sp CAG 257 and *R. gnavus*, and a depletion of beneficial species (e.g. *B. longum* and *F. prausnitzii*) linked to a healthy microbiome. *Blautia* sp CAG 257 has also been implicated in IBD and was recently identified as a predictor for Crohn's disease.^29^ Interestingly, taxa that were highly abundant were overall reduced in BAD, but taxa that were of low abundance were often elevated in BAD compared to healthy controls. This finding could be attributed to the selective environment in the colon of BAD patients, which may favor the growth of bile acid-tolerant bacteria such as *Lachnoclostridium scindens*, formerly known as *Clostridium scindens* (*C. scindens*)^10^ although, this species was only detected in 19% of our BAD patients.

In the functional analysis, the relative abundance of genes involved in bile acid transformation (*baiA*, *baiB,* and *hdhA*) and transportation was elevated in stool samples from BAD patients. The function of these genes provides insight into the bile acid pool of BAD patients. For instance, *baiA* encodes 3α-hydroxysteroid dehydrogenase, which catalyzes both the oxidation and reductive steps in bile acid metabolism involved in iso- and allo-bile acid production,^30^^,^^31^ while *baiB* encodes bile acid CoA-ligase, initiating the 7α-dehydroxylation pathway that produces the secondary bile acids DCA and LCA.^32^ Finally, *hdhA* encodes 7α-hydroxysteroid dehydrogenases, enzymes responsible for converting CDCA to UDCA.^33^^,^^34^

The elevated relative abundance of genes encoding hydroxysteroid dehydrogenases consistently reflects the higher levels of ketone (oxo-) and epimeric (iso-) bile acids observed in the stool of BAD patients. These dehydrogenation enzymes are responsible for the oxidation of bile acids, leading to the formation of ketones, as well as their reduction and often epimerization. This shift in bile acid composition highlights the distinct metabolic alterations occurring in BAD patients, in particular the reduction of allo-(5α) bile acids such as iso-allo-LCA acid. Further work will be needed to determine the precise changes.

The conversion of primary bile acids to secondary bile acids, LCA and DCA, requires the uptake of free primary bile acids into bacterial cells via proton-dependent bile acid transporters. Bacterial species capable of 7α-dehydroxylation possess this transporter.^35^ Several species have been identified to carry these genes, notably *R. gnavus* and various *Clostridium* species, such as *Clostridium innocuum* and *C. scindens*.^10^

The absence of a statistically significant difference in the relative abundance of *bsh* genes between the study groups suggests that bile salt hydrolase activity is not a distinguishing factor between BAD patients and healthy individuals. This finding aligns with the widespread distribution of *bsh* genes, with approximately 26% of bacterial strains in the Human Microbiome Project reference genome are reported to encode BSH.^36^ Given the crucial role of BSH enzymes in the deconjugation of bile acids—the initial step leading to the production of unconjugated bile acids, which in turn precedes the formation of secondary bile acids DCA and LCA—the similarity in *bsh* gene abundance across groups implies that other factors may be more influential in driving the differences in bile acid metabolism seen in BAD patients. Further investigation into downstream pathways, such as 7α-dehydroxylation, may be necessary to fully understand the microbial contributions to bile acid dysregulation.

Our findings show that genes involved in bile acid transformation are more strongly associated with the most severe forms of BAD (SeHCAT ≤ 4%) compared to moderate (SeHCAT 5%–8%) and mild (SeHCAT ≥ 9%) cases. These results support the hypothesis that alterations in bile acid me tabolism contribute not only to the presence of BAD but also to its progression, with more pronounced disruptions potentially linked to more severe disease.

Elevated levels of CDCA, a natural FXR agonist, were found in the stool samples of patients with BAD. Although CDCA activation of FXR decreases bile acid synthesis by stimulating FGF19 through FXR activation, other bile acids such as GCA, GCDCA, UDCA, GUDCA, and 7-oxo-DCA may inhibit FGF19 synthesis and increase bile acid synthesis.^14^^,^^37^^,^^38^ It is possible that the increased levels of antagonist bile acids in BAD patients, observed in our results, perpetuate the ongoing increase in bile acid synthesis and exacerbate the diarrhea associated with the disease.

The correlation between SeHCAT retention scores and specific bile acids, particularly the negative association with primary bile acids such as CA|UCA and CDCA, suggests impaired bile acid reabsorption, a characteristic of BAD, leads to an increased presence of these primary bile acids in the gut lumen, contributing to the pathophysiology of diarrhea. Conversely, the positive correlation between secondary bile acids and their derivatives suggests an adaptive or pathological shift in bile acid metabolism.

A major finding in this work is the association of genes related to biofilms in BAD patients. Biofilms are microbial communities of various populations of microorganisms.^39^ These form a matrix of extracellular polymeric substances attached to host surfaces.^40^ Baumgartner and colleagues found that IBS patients have significantly higher levels of biofilms in the ileum and right-sided colon compared to healthy individuals.^17^ Other studies of have found that bile acids can induce biofilm formation in bacterial species found in the human gut.^41^, ^42^, ^43^, ^44^ Our finding of elevated functional genes (*bssS*, *pgaA*, *pgaB*, *pgaC* and *pgaD*) related to biofilm formation in BAD patients indicates a role for biofilms in the pathogenesis of BAD. The formation of bacterial biofilms is a complex process that involves multiple proteins, which play different roles in the formation of a structured bacterial community. Among these proteins, BssS responds to environmental signals and regulates the expression of the functional genes.^45^ PgaA, PgaB, PgaC, and PgaD are involved in the synthesis of PGA, which is a major component of the extracellular matrix.^46^^,^^47^

In the same study, an increase of *E. coli* and *R. gnavus* and a reduction in *F. prausnitzii* were found in IBS patients who had biofilms in their ileum.^17^ Other studies of patients with IBS have also shown elevated levels of *E. coli* and *R. gnavus*,^48^ and a reduction in levels of *F. prausnitzii*.^49^^,^^50^ Indeed, *R. gnavus* can form biofilms *in vitro*,^17^ while the ability of *E. coli* to form biofilms *in vitro* varies depending on the growth conditions and the strain.^51^ Taken together, these observations form the basis of our hypothesis that biofilms in the ileum of BAD patients are associated with the presence of *R. gnavus* and could thus exacerbate their condition. Biofilms may present a physical barrier for bile acid reabsorption, in turn leading to excessive amounts of bile acids in the colon, altered feedback regulation, and diarrhea. This hypothesis may provide insight into some of the key features of BAD, including low FGF19, high fecal bile acids, low SeHCAT scores, and minimal inflammation.

Moreover, the significant correlation between *R. gnavus* and various bile acids highlights the interplay between this microbe and bile acid metabolism. *R. gnavus* may play a pivotal role in the biotransformation of bile acids, particularly by preferentially influencing their sulfation and dehydroxylation. This selective modulation has important implications for bile acid solubility, reabsorption, and the overall dynamics of enterohepatic circulation, thereby contributing to the maintenance of a new bile acid homeostasis in the gut of BAD patients. The lack of significant correlations between *E. coli* or *F. prausnitzii* and any bile acids in this study suggests that these species may have a less direct or no role in the dysregulation of bile acid metabolism associated with BAD.

An important limitation of our study is that the numbers of patients in the 2 groups were relatively small, although these were sufficient to give the significant findings we report. Another limitation is that a SeHCAT-negative IBS control group was not included, but the significant correlations we found between more severe SeHCAT retention values and our novel data help to address this. Interestingly, another study presented in abstract form has also found correlations between the abundance of *R. gnavus* and fecal bile acid concentrations in patients with IBS-D.^52^

## Conclusion

These findings underscore the nuanced interactions between the gut microbiome and bile acids, with *R. gnavus* emerging as a key player in the modulation of bile acid profiles. We report the changes in the gut microbiome composition in BAD patients are associated with disease severity. We provide evidence that the formation of bacterial biofilms in the ileum may contribute to the impaired bile acid reabsorption seen in idiopathic PBAD. While further investigation is needed to fully elucidate the role of biofilms, our observation may offer a new avenue for diagnosing BAD.
