# Supplementary material for: Ruminococcus gnavus and Biofilm Markers in Feces From Primary Bile Acid Diarrhea Patients Indicate New Disease Mechanisms and Potential for Diagnostic Testing
Source: Gastro Hep Adv. 2025 May 27;4(9):100712. doi: 10.1016/j.gastha.2025.100712 (PMC12702444; doi:10.1016/j.gastha.2025.100712)
Supplement: Supplementary Material [file mmc1.docx]

**Supplementary Materials**

**Supplementary Methods**

***Bile Acid Composition Analysis***

Bile acids were extracted from stool using a methanol extraction method adapted from Lin *et al*. (2018).^1^ In brief, the protocol involved sequential extraction steps with mixing and centrifugation. Stool samples (100 mg) were suspended in ice-cold water, vortexed, and centrifuged at 15,000 x g for 15 minutes at 4°C. After transferring the supernatant, methanol was added to the remaining pellet, followed by further vortexing and centrifugation. The final combined supernatant was collected carefully to avoid disturbing the pellet.

Study samples were prepared by aliquoting 40 µL of filtered fecal extracts onto a 96-well plate and adding 80 µL of LCMS grade methanol and 40 µL of IS solution mix (**Table 1**). The plate was mixed for two minutes on a plate mixer (800 rpm at 2-8 °C), then centrifuged at 3486 x g for 10 minutes at 4°C. Aliquots of 125 µL of clear supernatants of each sample were carefully transferred to an analytical 96-well plate which was then heat sealed, centrifuged at 3486 x g for 1 minute at 4°C and placed into the autosampler maintained at 4°C for the analysis. A pooled study reference (SR) sample was created by mixing equal parts of all the fecal extracts and prepared as the study samples. The pooled SR sample, analyzed throughout the run every 10 study samples, was used as quality control (QC) of the untargeted high-resolution mass spectrometry (HRMS) profiling data and to monitor assay performance. To assess the linearity response of the extracted features, a series of SR sample dilutions (100%, 80%, 60%, 40%, 20%, 10%, 1%) were created and analyzed at the start and end of the run.^2,3^ HRMS semi data were processed using TargetLynx application package within Masslynx software. Calibration curve solutions, consisting of ten concentration levels, and three sets of quantitative QC samples, at five concentration levels, were added to the plate and prepared as the study samples aliquoting 40 µL of the standard working solutions mixes of the semi-quantified bile acids instead of the samples. The quality of the semi-quantitative bile acids analysis was controlled using the quantitative QC samples (acceptance criteria of less than 20% deviation) and a coefficient of variation in SR samples lower than 30%. The advantages of semi-targeted LC-MS metabolic profiling, which allows accurate measurement of concentrations of selected panels of metabolites and simultaneous global profile data mining and metabolite discovery from a single sample injection, have been recently acknowledged.^4^

For relative abundance (area under the chromatographic peak) results, which were annotated using reference chemical standards analyzed at the beginning and end of the fecal samples run, the authentic chemical standards were split into mixes, to separate isomeric bile acid species, and also spiked into a subaliquot of the pooled study reference (SR) sample. This helped monitor any potential retention time shifts and to determine the retention time windows (regions of interest) used as an input, together with *m/z* values, for the targeted extraction and integration of the annotated bile acids using the R package peakPantheR.^5^ Features with a coefficient of variation greater than 30% in SR samples and a Pearson correlation with dilution factor below 0.7 were discarded.^6^ Pooled SR and spiked SR samples were prepared as the study samples.

The analysis was performed using ultra high-performance liquid chromatography (UHPLC)-HRMS, following the protocol established by Sarafian *et al*. (2015).^7^ In summary, data was acquired on an ACQUITY UHPLC coupled to a Xevo G2-S Q-ToF mass spectrometer with an electrospray ionization source operating in negative ion mode (Waters, Manchester, UK). Samples (2 µL) were injected onto an ACQUITY BEH C8 column (1.7 μm, 100 mm × 2.1 mm) maintained at 60°C. A gradient was applied consisting of 10:1 water:acetonitrile, 1 mM ammonium acetate, pH 4.15 (A) and 1:1 isopropanol: acetonitrile (B). MS parameters were as follows: capillary voltage 1.5 kV, cone voltage 60 V, source temperature 150 °C, desolvation temperature 600 °C, desolvation gas flow 1000 L/h, and cone gas flow 150 L/h. Masslynx V4.2 software (Waters, Manchester, U.K.) was used for data acquisition and visual inspection.

***DNA Sequence Quality Control***

Following sequencing, paired-end sequencing, were generated. Quality control checks were executed using the bioinformatics tools FastQC and MultiQC on the raw sequenced data [184], [185]. Specifically, the mean quality score for each base position in the reads were examined before and after trimming and filtering. Adapter-trimming, quality-related trimming, quality filtering and length filtering were carried out using the program BBDuk version 37.62 which is part of BBTools [186].

Following sequencing, paired-end reads were generated and subjected to rigorous quality control using the bioinformatics tools FastQC and MultiQC on the raw sequence data [184], [185]. Quality assessment involved examining the mean quality score in the reads before and after trimming and filtering. Adapter trimming, quality trimming, and length filtering were conducted using BBDuk, a component of the BBTools suite [186]. The following parameters were used:

1. ordered=t - this output reads in same order as input
2. ktrim=r -once a reference kmer is matched in a read, that kmer and all the bases to the right will be trimmed
3. k=21 -specifies the kmer length as 21bases
4. mink=10 -"mink" allows it to use shorter kmers (10 bases) at the ends of the read
5. hdist=2 -number of permitted mismatches
6. qtrim=r -quality trim on right
7. trimq=20 -1 in 100 or 99%
8. minlen=50 -discard reads shorter than 50bp after trimming
9. maq=10 #This will discard reads with average quality below 10 specifically, 90%

For assembly, host DNA was removed by filtering human reads with BBMap version 37.62, using the GRCh38 human genome reference, yielding a dataset optimized for downstream microbial analysis.

***Analysis of Bile Acid Transforming Genes***

HUMAnN (version 3.0.3) pipeline was used to profile the abundance of microbial metabolic genes from metagenomic sequencing data [187], [188]. Default settings were applied, which profiled microbial communities with MetaPhlAn (version 3.1) using unique clade-specific marker genes for taxonomic identification; then mapped reads to ChocoPhlAn pangenome database (mpa_v30_CHOCOPhlAn_201901) which contains functionally annotated species using Bowtie2 to produce a species-specific list of functional genes. Employing UniRef90 protein reference database, sequences were screened using DIAMOND open-source algorithm that placed reads into protein families using PFam. To facilitate comparisons between samples with different sequencing depths, the subsequent protein family files were all normalized to copies per million (CPM) using the built-in ‘humann_renorm_table’ script. The separate sample files were combined before being analyzed. The functional bacterial genes responsible for transforming bile acids from primary to secondary bile acids were analyzed to determine the transforming potential.

**References**

1. Lin CY, Zhao L, Huang T, et al. Spexin acts as novel regulator for bile acid synthesis. Front Physiol 2018;9:1-14.

2. Sands CJ, Gómez-Romero M, Correia G, et al. Representing the Metabolome with High Fidelity: Range and Response as Quality Control Factors in LC-MS-Based Global Profiling. Anal Chem 2021;93:1924-1933.

3. Mullish BH, Martinez-Gili L, Chekmeneva E, et al. Assessing the clinical value of faecal bile acid profiling to predict recurrence in primary Clostridioides difficile infection. Aliment Pharmacol Ther 2022;56:1556-1569.

4. Amer B, Deshpande RR, Bird SS. Simultaneous Quantitation and Discovery (SQUAD) Analysis: Combining the Best of Targeted and Untargeted Mass Spectrometry-Based Metabolomics. Metabolites 2023;13.

5. Wolfer AM, Correia GDS, Sands CJ, et al. peakPantheR, an R package for large-scale targeted extraction and integration of annotated metabolic features in LC-MS profiling datasets. Bioinformatics 2021;37:4886-4888.

6. Sands CJ, Wolfer AM, Correia GDS, et al. The nPYc-Toolbox, a Python module for the pre-processing, quality-control and analysis of metabolic profiling datasets. Bioinformatics 2019;35:5359-5360.

7. Sarafian MH, Lewis MR, Pechlivanis A, et al. Bile Acid Profiling and Quantification in Biofluids Using Ultra-Performance Liquid Chromatography Tandem Mass Spectrometry. Anal Chem 2015;87:9662-9670.
